# Exploring Host–Commensal Interactions in the Respiratory Tract

**DOI:** 10.3389/fimmu.2017.01971

**Published:** 2018-01-17

**Authors:** Sudhanshu Shekhar, Karl Schenck, Fernanda Cristina Petersen

**Affiliations:** ^1^Faculty of Dentistry, Department of Oral Biology, University of Oslo, Oslo, Norway

**Keywords:** host, commensal, immunity, lungs, vaccine

## Abstract

Commensal microbes are currently in the limelight in biomedical research because they play an important role in health and disease. Humans harbor an enormous diversity of commensals in various parts of the body, including the gastrointestinal and respiratory tracts. Advancement in metagenomic and other omic approaches, and development of suitable animal models have provided an unprecedented appreciation into the diversity of commensals, and the intricacies of their intimate communication with the host immune system. Most studies have focused on the host–commensal interaction in the gut, while less is known on this relationship in other sites of the body, such as the respiratory tract. In this article, we review emerging data from human and animal studies on the host responses to respiratory commensals, immune cross-reactivity between commensals and pathogens, and use of commensals as a vaccine delivery system. A better understanding of the delicate interplay between commensals and host may aid in efforts to develop effective vaccines and therapeutics.

## Introduction

Microbial commensalism is traditionally defined as a microbe–host relationship in which microbes benefit from the host, but the latter remains largely unaffected. The commensal microbiota has co-evolved with humans for eons and effectively colonizes various body sites, including the intestinal and respiratory tracts. With the advent of new experimental tools, it is becoming clearer that this commensal–host relationship is much more complex and sophisticated than previously thought. Metagenomic and other omic approaches and the use of germ-free and gnotobiotic animals have provided cutting-edge evidence that gut commensals exert a profound impact on the overall health of the body that is crucial for maintaining homeostasis between the host and commensals. These commensals are not only endowed with unique roles in food digestion, immune system development, production of vitamins and hormones, but also in the production of neurotransmitters/neuromodulators, and development of the central nervous system (1–3). Dysbiosis, the disruption of host–commensal homeostasis, can result in a large array of human diseases, such as obesity, diabetes, allergy, and inflammatory bowel disease (1, 2). Of note, some of the commensals with high pathogenic potential are termed as commensal pathogens or pathobionts (4). The contribution of gut commensals in health and disease is widely studied. Limited information is, however, available on the role of commensals colonizing the respiratory tract, which harbors a complex microbial community subjected to continuous exposure from the external environment. Recent studies using humans and mice have begun to shed light on the functional roles played by respiratory commensals in immunity and immunomodulation (5–12). Data are also accumulating on the potential of respiratory commensals in designing novel prophylactic strategies due to their ability to act as a vaccine delivery system and to show immune cross-reactivity with pathogens (7, 13–17).

In this article, we review current literature on the interaction between host and commensal microbes in the respiratory tract. Specifically, we focus on host immunity to respiratory tract commensals and on the use of commensal microbes for prophylactic purposes. A better understanding of the cross-talk between commensals and host holds promise for developing effective and safe prophylactic and therapeutic strategies.

## The Respiratory Tract Microbiota

The respiratory tract consists of the upper respiratory tract (URT) and the lower respiratory tract (LRT). The major passages and structures of the URT extend from the nasal and oral cavities to the throat, whereas the LRT includes the trachea and the lung. Although gene sequencing using 16S/18S ribosomal RNA has enabled culture-independent characterization of the URT microbiota, relatively fewer studies have been conducted to study the LRT microbiota in healthy individuals (18). Colonization of the URT by commensals depends upon host and environmental factors, such as mode of child delivery (vaginal vs cesarean), dietary habits (breast milk vs formula), use of antibiotics, and to a lesser extent on host genetics (19, 20). Healthy full-term neonates in their first hours of life carry a large number of microbes in their respiratory tract that are considered to be of maternal origin. During and following the first week of life, predominant microbial genera colonizing the URT include *Staphylococcus, Corynebacterium*, and *Dolosigranulum*. This is followed by a later increase in *Moraxella* and *Streptococcus* (20, 21). Early colonization by *Corynebacterium, Dolosigranulum*, and *Moraxella* are particularly correlated with respiratory health (20). In healthy adult individuals, the nasal cavity and nasopharynx harbor a community of microorganisms represented by *Staphylococcus, Propionibacterium, Corynebacterium, Streptococcus, Dolosigranulum*, and *Moraxella*, while the oropharynx is mainly colonized by *Streptococcus, Rothia, Veillonella*, and *Prevotella* (21, 22). Commonly found fungal genera in the URT consist of *Aspergillus, Penicillium*, and *Candida*, whereas viral genera include Anellovirus and Herpesvirus (23, 24).

The LRT has long been thought to be sterile. The historical reasons for such an assumption were recently reviewed, and several arguments put forward strongly suggest that sterility of the lungs is a highly unlikely scenario (25). The premise upon which this assumption was installed encompasses microbiological assays that are culture-dependent, and that have been biased toward the identification of respiratory pathogens. With the help of next-generation sequencing, Hilty et al. for the first time reported the occurrence of a diverse microbial community in the lungs of healthy individuals (26). This has been followed by various studies using metagenomics that show the existence of a lung microbiota (18). As the composition of the commensal microbiota in the URT and LRT is similar, it has been maintained that the presence of a LRT microbiota merely stems from microaspiration and mucosal dispersion of microbes from the URT. Sample collection from the lung is challenging because pulmonary samples are liable to contamination by the URT microbiota while performing bronchoscopy. Recovery of low quantities of microbial biomass in the samples also poses challenges for microbiological analysis. In healthy adults, cough, mucociliary clearance, and host defenses contribute to keep a low number of microorganisms in the lungs. In respiratory diseases, this situation is changed to one in which conditions for microbial growth are favored, resulting in expansion of the microbiota (1, 25). These findings confirm the existence of the microbiota in the lung, but whether the microbiota is microaspirated or dispersed from the URT remains elusive. However, exclusive presence of certain species in the LRT, such as *Tropheryma whipplei*, supports the notion that microbes found in the LRT samples are, at least in part, not derived from the URT (27).

## The Respiratory Mucosal Immune System

The mucosal respiratory immune system provides a podium for immune reactions due to its proximity with the external environment. The mucosal layer is lined by ciliated pseudostratified columnar epithelium in the nasal cavity, larynx, trachea, and bronchi, whereas simple squamous epithelium is found in pulmonary alveoli. The epithelial layer is interspersed with mucus-producing goblet cells (G), which secrete mucus that prevents the entry of pathogens by entrapping them (28). The presence of microfold (M) cells is well-described in the gut-associated lymphoid tissue and mucosa-associated lymphoid tissue of the gastrointestinal tract where they transport antigens across the epithelial cell layer from the intestinal lumen to the lamina propria (29). In the respiratory epithelium, this is not firmly established. Previous studies have reported that pathogens like *Mycobacterium tuberculosis* and reovirus can gain access to the body from the murine lung *via* M cells (30, 31). Furthermore, Id2−/− mice that are deficient in lymphoid tissues reveal a similar frequency of M cells in the nasal epithelia and generate significantly higher antigen-specific antibody responses compared with Id2+/− control mice (32). These results indicate that respiratory M cells represent an alternative gateway for antigen transport and sampling in mice. On the other hand, although the occurrence of isolated lymphoid follicles localized in contact with bronchial epithelium in children indicates the presence of cells in the epithelium that have an M cell-like function, further studies are required to arrive at a definitive conclusion about M cells in humans (33). The epithelial layer confers physical resistance to the invading pathogens and produces antibacterial peptides, such as β-defensins and cathelicidins (34). The lymphoid tissues associated with the mucosal layer include nasopharynx-associated lymphoid tissue and bronchi-associated lymphoid tissue and contain a variety of immune cells, such as T cells, B cells, and dendritic cells (DCs) for induction, regulation, and effector function of mucosal immune responses (35, 36). In the respiratory tract, DCs are present beneath the epithelial layer and capture microbial antigens by projecting their dendrites through intercellular spaces (36, 37). DCs upregulate costimulatory molecules (CD40, CD80, CD83, and CD86), produce multiple cytokines (IL-12, IL-10, IL-23, IL-6, and IL-23), and migrate *via* afferent lymph vessels to the lung-draining mediastinal lymph nodes to present the captured antigens to naive T cells. T cell subsets, including CD4+ and CD8+ T cells are primed by DCs and transform into effector T cells that exit the lymph nodes *via* efferent lymph vessels and migrate to effector sites. CD4+ T cells perform their effector function by secreting cytokines such as IFN-γ, IL-10, and IL-17, whereas CD8+ T cells do so *via* granzyme/perforin and Fas–FasL pathway (37). After encountering their cognate antigens, B cells in the lymph nodes differentiate into plasma cells that produce antibodies like secretory IgA (38, 39).

## Host Immunity to Respiratory Commensals

Germ-free and gnotobiotic animals have proven to be critical tools to study the dynamic relationship of the immune system with the microbiota. Although most of the host responses induced by the microbiota have been attributed to microbial residents of the gut, depletion of the microbiota in germ-free animals is not restricted to the gut, but extends to all organs. Thus, attempts have been made to deplete the microbiota at specific sites to better understand the contribution of the specific microbiota in immunity and pathology. Unfortunately, efficient animal models to specifically study the respiratory tract microbiota are currently not available. The majority of the data on host–respiratory commensal interactions stems from *in vitro* studies involving bacterial commensals and immune cells isolated from humans and mice. Recent studies provide evidence that bacterial commensals have an impact on modulation of inflammatory responses and suppression/killing of pathogens in the respiratory tract (6, 8–12). Larsen et al. analyzed the phenotypic and functional changes in human DCs in response to respiratory bacterial commensals (*Prevotella* spp. and *Veillonella* spp.) and pathogens (*Actinomyces* spp.) (6). Upon stimulation with commensals, monocyte-derived DCs exhibited higher expression of CD40, CD80, and CD86 and enhanced production of pro- and anti-inflammatory cytokines (IL-12p70, IL-23, and IL-10) compared with the unstimulated DCs. The expression level of the costimulatory molecules on DCs was similar between the commensals and pathogens, whereas commensals produced 3–5 times lower levels of cytokines compared with the pathogenic bacteria (6). Addition of *Prevotella* spp. into cultures of DCs pulsed with the pathogenic species *Haemophilus influenzae* partially reduced the production of IL-12p70, but not of IL-23 and IL-10, by the DCs (6). This indicates that *Prevotella* spp. can suppress Th1 immunity specific to *H. influenzae*.

A recent study evaluated the impact of commensals, including *Streptococcus mitis, Streptococcus salivarius, Streptococcus gordonii*, and *Streptococcus sanguinis* on pro-inflammatory responses in oral keratinocytes by the pathogens, *Aggregatibacter actinomycetemcomitans*, and *Fusobacterium nucleatum* (12). The production of IL-8, a chemokine that induces chemotaxis in granulocytes, was significantly higher in human epithelial cell lines infected with the pathogenic bacteria than by the cells exposed to commensals. Upon co-infection with both commensal and pathogenic bacteria, the commensals reduced the pathogen-induced IL-8 secretion (12). Similar modulation of the IL-8 response by *S. salivarius* strain K12 was demonstrated in relation to the pathogen *S. pyogenes* (9). More importantly, intragastric administration of live *S. salivarius* strain JIM8772 led to a significant reduction in intestinal inflammatory reactions in a mouse model of TNBS-induced colitis, compared with control buffer (11). This suggests an immunomodulatory and protective role for this commensal in inflammatory conditions. The mechanisms by which commensals exert their effect is yet to be elucidated. Overall, these data indicate that commensal bacteria modulate host responses induced by pathogens, which is in line with previous studies showing a positive impact of using *S. salivarius* strains as probiotics against streptococcal pharyngitis and halitosis (40). Anti-inflammatory properties of commensal bacteria have the potential to be used to treat inflammatory and autoimmune diseases.

Little is known about the interaction of the host with commensal viruses and fungi that inhabit the respiratory system. Latent infection with herpesviruses can lead to opportunistic infections in immunocompromised individuals (41). Recent findings, however, highlight a new role for these viruses in increasing host resistance to bacterial infections. Infection with herpesviruses in mice results in chronic production of large quantities of IFN-γ and activation of macrophages that confer protection from subsequent infection with *Listeria monocytogenes* and *Yersinia pestis* (42). On the other hand, the opportunisitic fungus *Aspergillus fumigatus* causes severe infections in patients with a compromised immune system (43). *Aspergillus* spp. first encounter alveolar macrophages in the lung and are cleared by them *via* an NADPH oxidase-dependent pathway (44). A strong Th1 response is central to protective immunity against *A. fumigatus* infection, but the role of IL-17 in immunity to aspergillosis is poorly understood (45). *In vivo* neutralization of IL-17 in non-immunocompromised mice led to increased fungal loads in the lungs when subjected to *A. fumigatus* infection, suggesting a protective role for IL-17 (46). To understand the contribution of IL-17 in humans, Chai et al. examined the IL-17 response to fungal infection using human peripheral blood mononuclear cells (PBMCs) and clinical samples from patients with invasive aspergillosis (47). Incubation of live *A. fumigatus* with human PBMCs resulted in enhanced Th1, but not Th17, responses compared with the PBMC culture without fungal stimulation. This finding was in line with the low levels of IL-17 in the bronchoalveolar lavage fluid and serum of patients with invasive aspergillosis (47). Thus, in contrast to mice, human immunity to *A. fumigatus* does not appear to be dependent on IL-17 responses. There is a need to know more about the immune responses to these commensals to determine if they are to be used for prophylactic purposes, which is important for developing vaccines against opportunistic infections in patients with compromised immune system.

## Cross-Reactivity Between Commensals and Pathogens

**Figure 1:**
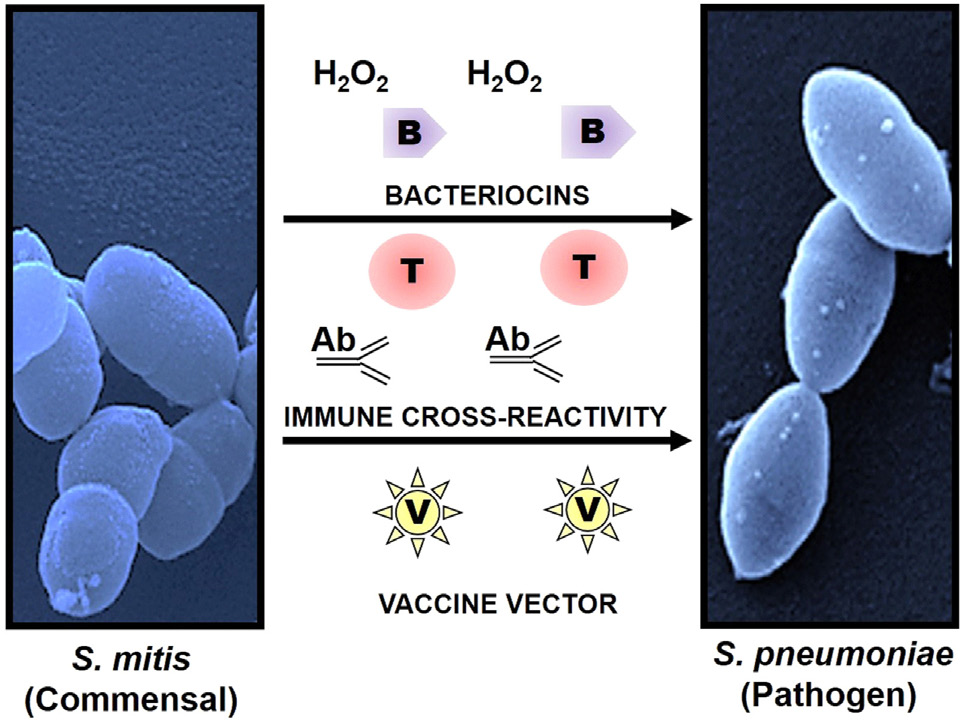
Use of commensals as a prophylactic strategy against respiratory pathogens. Commensals showing high homology with pathogenic species have the potential to induce cross-reactive immune responses and to act as a vaccine delivery system. Commensal *Streptococcus mitis* induces cross-reactive antibody (Ab) and T cell (T) responses against pathogen *Streptococcus pneumoniae*. Production of bacteriocins (B) and hydrogen peroxide (H_2_O_2_) by *S. mitis* elicits direct protection against pathogens like *S. pneumoniae*. In addition, *S. mitis* has been used as a potent vector (V) for delivery of heterologous bacterial and viral antigens, which confers robust and antigen-specific immunity against respiratory infections. Scanning electron microscopy images of *S. mitis* NCTC12261 (left), and *S. pneumoniae* TIGR4 (right).

A high degree of antigenic similarity between phylogenetically related commensal and pathogenic bacteria supports the hypothesis that natural immunity against pathogens in most adults is the outcome of repeated colonization by commensals that share epitopes during childhood and youth. This hypothesis is anchored on various studies that have demonstrated serological and cell-mediated cross-recognition between respiratory commensals and pathogens, particularly commensal streptococci and *Streptococcus pneumoniae* (7, 15–17, 48–52) (Figure 1). Recently, Skov Sorensen et al. performed a comprehensive analysis of genetic and antigenic similarities between a range of commensal streptococci and *S. pneumoniae* and found that 74% of 66 commensal *S. mitis* strains produced capsule *via* the Wzy/Wzx pathway, which is in contrast with the previous assumption that capsule production by *S. pneumoniae* distinguishes them from commensal streptococci that lack capsule (15). Furthermore, double immunodiffusion experiments showed that rabbit antisera raised with different strains of *S. mitis* and *S. oralis* exhibited cross-reactivity with capsular polysaccharide antigens of *S. pneumoniae* (15). These findings raise significant questions regarding misidentification of *S. pneumoniae* due to their capsular similarity with commensal streptococci, which can have consequences for effective vaccination in infants and elderly. Moreover, it is worth exploring the immune responses against *S. mitis* and its relationship with *S. pneumoniae* in humans. Studies have shown that salivary IgA antibodies in infants react to *S. mitis* antigens, but it is unclear whether the antibodies are elicited by *S. mitis* or by cross-reactive streptococcal pathogens (53–55). Ongoing studies in our laboratory focus at the antibody-mediated cross-reactivity between *S. mitis* and pneumococcal antigens. In addition, commensal *Neisseria lactamica* spp. and pathogenic *Neisseria meningitidis* have been reported to share major outer membrane cross-reactive antigens (16, 17). Future studies need to assess whether shared immune reactivity between respiratory commensals and pathogens, e.g., *S. mitis* and *S. pneumoniae*, could be translated into cross-protection against pathogenic *S. pneumoniae*. Immune cross-reactivity is not confided to antibodies. T cells specific to commensals can cross-react with pathogens as well. Recently, we examined the phenotype and function of human memory CD4+ T helper (Th) cells reactive with antigens from *S. mitis* by isolation of memory CD4+ Th cells from healthy human donors and construction of T cell libraries (7). Our findings demonstrated that Th17 cells that were isolated on the basis of their reactivity with *S. mitis* antigens also responded to pneumococcal antigens (7). Since Th17 cells play an important role in clearance of *S. pneumoniae, S. mitis*-specific Th17 cross-reactivity with *S. pneumoniae* might be used as a way forward to devise a prophylactic strategy against pneumococcal infections.

It may not be out of place to mention here that commensals, such as *S. mitis*, can exert direct protection against respiratory pathogens, including *S. pneumoniae*, by producing hydrogen peroxide (H_2_O_2_) and bacteriocins that have an inhibitory effect on the pathogens (12, 56) (Figure 1).

## Commensals as a Vaccine Delivery System

Some mucosal vaccines, such as polio vaccine, elicit antigen-specific immune responses against a variety of pathogens (57, 58). Since commensals and pathogens colonize the same mucosal surfaces in the respiratory tract, commensals have gained interest as a vaccine delivery system for combating respiratory infections. Previous studies have tested the efficacy of commensal bacteria such as *S. gordonii* and *Lactobacillus lactis* as vectors of antigen delivery due to their commensal nature and ease in genetic manipulation. While *S. gordonii* offers a platform to express various heterologous antigens (e.g., diphtheria toxin antigens), it fails to mount a robust protective response against the recombinant antigens (59, 60). Similarly, a number of mouse studies using *L. lactis* have been conducted to examine the expression of heterologous antigens and to analyze antigen-specific immune responses (61). Intranasal immunization of mice with recombinant *L. lactis* expressing human papillomavirus type 16 (HPV-16) E7 protein elicited antigen-specific T cell responses characterized by IFN-γ compared to the non-immunized mice or mice with wild-type *Lactobacillus* (61). Overall, these commensals have shown a potential as vaccine delivery vehicles, but their ability to confer robust protective immunity is limited. This might be due to poor colonizing capacity of *Lactobacillus* and low abundance of *S. gordonii* (62, 63). Two recent studies have evaluated the potential of *S. mitis* as a vaccine vector for *M. tuberculosis* and human immunodeficiency virus (HIV) antigens (13, 14). Oral administration of recombinant *S. mitis* expressing HIV envelope protein in germ-free mice resulted in effective and persistent colonization and was associated with strong antigen-specific IgA and IgG, but non-responsive T cell, responses (14). Similar colonization and antibody responses (IgA and IgG) were observed when gnotobiotic piglets received *S. mitis* recombined with *M. tuberculosis* protein (Ag85b) through the oral route (13). These findings suggest that *S. mitis* has the potential to function as a vaccine vector, which may be attributed to its persistent colonization, abundance in the oral cavity, stable expression of the genes encoding vaccine candidates, and robust antibody responses (Figure 1). However, it has to be noted that these studies have been conducted in germ-free or gnotobiotic animals that may not mimic what happens under natural conditions and in humans, thus warranting the use of animal models with intact microbiota to arrive at a more realistic conclusion.

## Conclusion and Future Perspectives

With advancement in sequencing techniques, it is now possible to critically examine and analyze various microbiological and immunological aspects of respiratory tract commensals. From the microbial side, studies that move from the identification of genus or species to include the collection of genes (metagenomics) or transcripts (metatranscriptomics) in the microbiota are likely to contribute to a better understanding of their role in health and disease, and to reveal possible targets for intervention. To date, metagenomic and metatranscriptomic results are still limited to few studies (1, 64). In respect to host–microbial interactions, recent studies have shown that respiratory commensals modulate host immunity by suppressing proinflammatory responses elicited during infections and autoimmune diseases. Controlling excessive immune responses is beneficial to the host as uncontrolled responses can be pathological. On the flip side, immune suppression can make the host prone to infections. Therefore, a delicate balance between host response and microbial activity is critical for maintaining homeostasis in the body. Furthermore, immune cross-reactivity between commensals and pathogens has the potential to generate protective immunity against pathogens. Future studies are required to address the following questions: (1) Can immune cross-reactivity between commensals and pathogens be translated into cross-protection against pathogens? (2) What are the cross-reactive antigens that can be used as potential vaccine candidates? (3) What are the specific agonists produced by commensals to activate different immune cells? (4) How can we develop animal models that specifically lack respiratory tract microbiota? (5) Does an alteration in composition and/or function of the respiratory microbiota lead to the development of respiratory diseases? (6) Can the therapeutic manipulation of the respiratory microbiota be used as a tool to curb diseases? Answers to these questions can lead to better designing of vaccines and drugs against respiratory pathogens.

## Author Contributions

SS is the primary author of this manuscript. FP and SS produced the included figure. All authors assisted in the conception of this review, acquisition of relevant literature, and editing the manuscript. All authors gave approval of the final version to be published.

## Conflict of Interest Statement

The authors declare that this work was conducted in the absence of any commercial or financial relationships that could be construed as a potential conflict of interest.
